# Comparison Between Adenoid-Nasopharynx Ratio and Endoscopic Examination of Adenoid Hypertrophy: A Systematic Review and Meta-Analysis

**DOI:** 10.7759/cureus.89452

**Published:** 2025-08-05

**Authors:** Rajesh Gupta, Manas Ranjan Rout, Sabita Rauniyar, Subrat k Patra, Sanghamitra Jena

**Affiliations:** 1 Otolaryngology — Head and Neck Surgery, Kalinga Institute of Medical Sciences (KIMS), Bhubaneswar, IND; 2 Orthodontics and Dentofacial Orthopaedics, Om Shanti Dental Clinic Pvt. Ltd., Kathmandu, NPL; 3 Orthodontics and Dentofacial Orthopaedics, Kalinga Institute of Dental Sciences (KIDS), Bhubaneswar, IND

**Keywords:** adenoid hypertrophy, adenoid-nasopharyngeal ratio (anr), endoscopic methods, lateral neck radiography (lnr), otitis media

## Abstract

Otitis media is a major health issue that usually results from adenoid hypertrophy. Diagnosis is based on symptoms, such as mouth breathing, and imaging studies, including lateral neck radiography (LNR). The adenoid-nasopharyngeal ratio (ANR) is one of the most important and widely used criteria in LNR studies. It helps estimate the real size of adenoid gland measurements. The present systematic review aimed to detect the accuracy of adenoid hypertrophy diagnosis by LNR and compare the same with the endoscopic method.

According to the Preferred Reporting Items for Systematic Reviews and Meta-Analyses for Diagnostic Test Accuracy (PRISMA-DTA) guideline, a scoping evaluation of the articles was performed. The electronic databases considered were PubMed, Scopus, Web of Science, and Embase, from January 2004 to November 2024. The inclusion and exclusion criteria were applied. A total of 20 articles were studied in detail, 13 full-text articles were further excluded, and finally, seven articles were found appropriate to be included in this review. The outcome of this systematic review has led to the conclusion that endoscopic examination, when employed for measurement of adenoid hypertrophy, has shown extremely positive and promising results as compared to cephalometric and head-neck radiographs.

## Introduction and background

Adenoids are masses of lymphoid tissue located in the upper part of the throat behind the nose, which play a role in the immune system by trapping pathogens that enter the nose [1]. Adenoid hypertrophy is the abnormal enlargement of lymphoid tissue located in the nasopharynx. This can block the eustachian tube, leading to the development of middle ear infections such as otitis media, particularly in pediatric populations [2]. However, when they become enlarged (hypertrophied), they can obstruct the nasal airway and cause various symptoms, especially in children, who are more commonly affected due to the natural size of the adenoids in early childhood [3,4]. Adenoid hypertrophy can be caused by recurrent infections, allergies, or inflammation, leading to chronic issues such as obstructive sleep apnea in some cases. The condition can occur independently or coexist with other conditions like tonsillar hypertrophy [5,6].

Accurate diagnosis of adenoid hypertrophy is crucial for managing associated conditions, such as recurrent otitis media, obstructive sleep apnea, and chronic nasal obstruction, especially in children. This diagnosis can guide decisions regarding surgical intervention, including adenoidectomy [7]. Traditionally, two diagnostic tools are used: lateral neck radiographs (LNRs), which measure the adenoid-nasopharynx ratio (ANR), and endoscopic examination of the nasopharynx, which provides a direct visual assessment. Treatment options range from medical management (e.g., intranasal topical steroids) to surgical removal of the adenoids (adenoidectomy) in more severe or chronic cases [8].

This review aimed to compare the efficacy of the ANR and nasopharyngeal endoscopy for diagnosing adenoid hypertrophy. By examining their diagnostic accuracy, clinical utility, and limitations, this article aims to provide a clearer understanding of when each tool should be used in practice.

## Review

Materials and methods

This systematic review followed the protocols set by the Preferred Reporting Items for Systematic Reviews and Meta-Analyses for Diagnostic Test Accuracy (PRISMA-DTA) and is registered in the National Institutes of Health database (Prospective Register of Systematic Reviews (PROSPERO) registration no. CRD42024581427). The review was conducted using the 'population intervention comparator outcome' (PICO) format. Studies featuring patients with nasopharyngeal cephalograms involving oral and maxillofacial structures and endoscopic measurement of adenoid hypertrophy and the correlation of ANR were identified.

Inclusion and Exclusion Criteria

Prospective or retrospective cohort studies; randomized controlled trials (RCTs); any type of comparison between LNR and the endoscopic method or approach; all reported outcomes related to LNR and the endoscopic method or approach; and articles published in English from January 2004 to November 2024 were included in the review. Case reports, case-control studies, personal opinions, letters to the editor, interviews, and proof of concept were excluded. The search strategy is detailed in Table 1.

**Table 1:** The PRISMA-DTA search strategy summary PRISMA-DTA: Preferred Reporting Items for Systematic Reviews and Meta-Analyses for Diagnostic Test Accuracy, MeSH: Medical subject headings, RCT: Randomized controlled trials

Search strategy	Particulars
Databases searched	PubMed, Embase, Scopus, Web of Science
Date of final search execution	November 15, 2024
Timeframe covered	January 2004 – November 2024
Language restrictions	English
Study types included	RCTs, cohort studies
MeSH example (PubMed)	("adenoid hypertrophy"[MeSH Terms] OR "adenoid enlargement"[Title/Abstract]) AND ("lateral neck radiograph"[Title/Abstract] OR "lateral radiograph"[Title/Abstract] OR "cephalogram"[Title/Abstract]) AND ("endoscopy"[Title/Abstract] OR "nasopharyngeal endoscopy"[Title/Abstract]) AND ("A/N ratio"[Title/Abstract] OR "adenoid nasopharyngeal ratio"[Title/Abstract])
Screening and selection	Performed independently by two reviewers; discrepancies resolved through consensus with a third reviewer.

Data Collection

To ensure the reproducibility and credibility of the review findings, a structured and standardized data extraction process was employed. Two independent reviewers (author RG and author MRR) performed data extraction using a pre-piloted, standardized form based on the PICO framework. Each reviewer independently extracted the following information from the included studies: study characteristics (authors, year, country); sample size; participant demographics; diagnostic methods (lateral neck radiography vs. nasopharyngeal endoscopy); measured outcomes; and statistical estimates (effect size, standard error). A third reviewer (author SR) resolved discrepancies by cross-checking the original articles. A double-entry method was employed to reduce transcription errors. Kappa statistics were calculated to measure inter-rater agreement during pilot testing, yielding a value of 0.82, indicating substantial agreement. All extracted data were verified before inclusion in the meta-analysis.

Results

Study Selection and Characteristics

**Figure 1:**
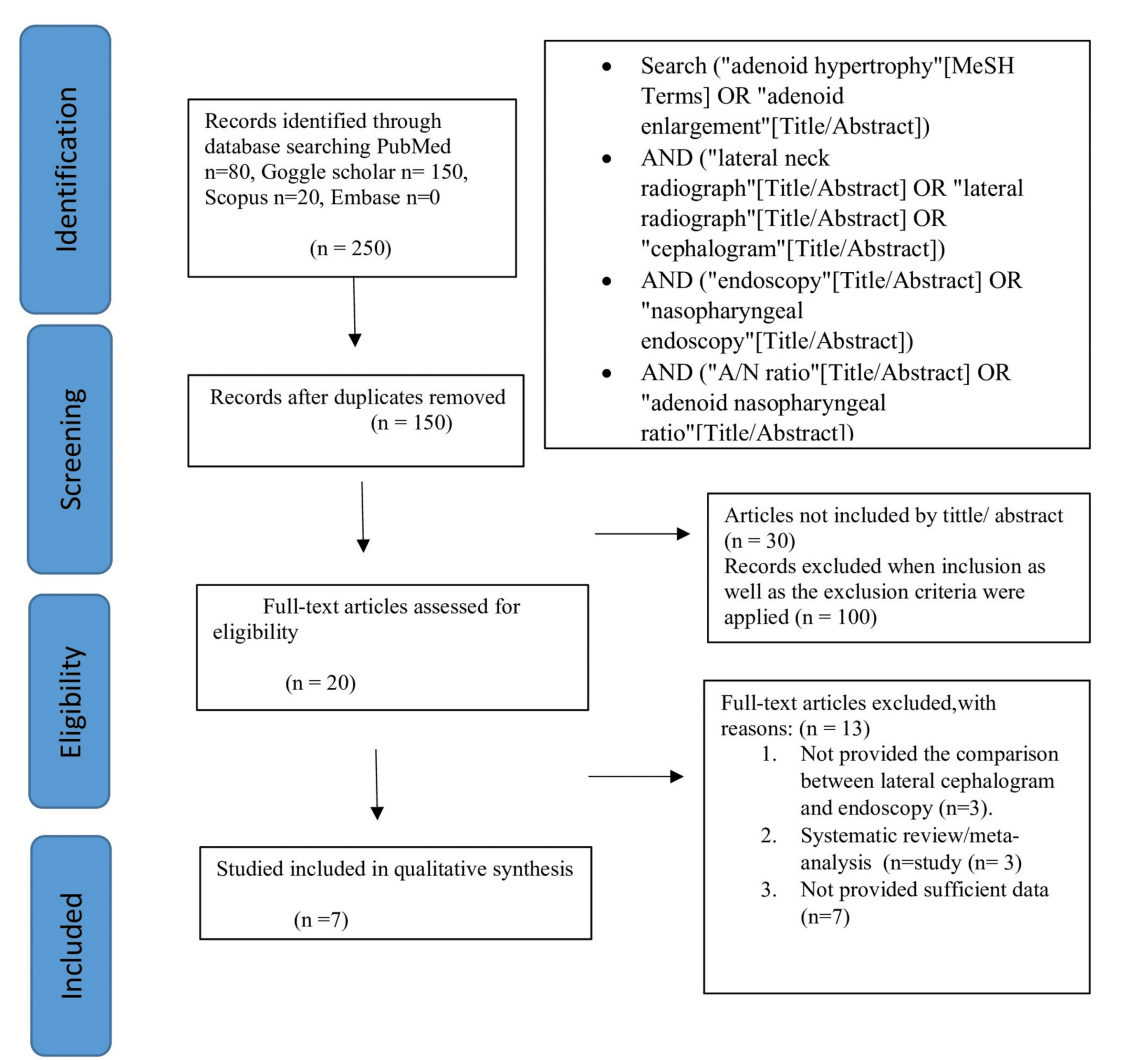
Flowchart of screening and selection of articles

The selection of studies for this review was carried out in a two-stage process. In the initial screening phase, 250 articles were shortlisted based on the relevance of their titles and abstracts to the review topic. Following this, 100 duplicate articles were identified and removed. Eligibility criteria were then applied to the remaining records, resulting in the exclusion of an additional 130 articles. Ultimately, 20 articles were retained for further evaluation. Upon detailed assessment, 13 full-text articles were excluded, with justifications documented. As a result, this review incorporates a final selection of seven studies that met the criteria for qualitative synthesis. A detailed flowchart illustrating the inclusion and exclusion process is provided in Figure 1.

The included studies were conducted across various countries, such as Turkey, Kano (Nigeria), Iran, Bangkok, and Indonesia, and span a publication period of two decades, from 2004 to 2024. A summary of key study characteristics is presented in Table 2.

**Table 2:** Brief description of the study characteristics ENE: Endoscopic nasal examination, LC: Lateral cephalogram

Authors	Year	Country	Evaluation method	Data source	Total sample	Lower age (in years)	Upper age (in years)	Correlation value (r-value)	ENE mean	ENE SD	LC mean	LC SD	Study design	Examiner
Cayalaki et al. [9]	2009	Turkery	ENE+LC	Baskent University	85	2	12	0.511 (p<0.0001)	88.5	12	0.87	0.1	Prospective	1
Saedi et al. [10]	2011	Iran	ENE+LC	Imam Khomeini Hospital	96	3	20	0.302 (p = 004	77.36	13.48	74.69	15.159	Prospective	1
Acar et al. [11]	2014	Turkey	ENE+LC	Dr. Sami Ulus Children’s Hospital, and the Department of Otorhinolaryngology, Head and Neck Surgery, Kecioren Training and Research Hospital	46	2	14	0.334 (p=0.023)	64.6	19.5	16.7	14.4	Prospective	1
Talebian et al. [4]	2018	Iran	ENE+LC	Mashhad University	27	4	13	0.46 (p=0.01)	19.15	5.33	18.69	3.29	Cross-sectional	1
Pisutsiri et al. [5]	2021	Bangkok	ENE+LC	Mahidol University	43	2	14	0.567 (p<0.001)	79.52	16.41	72.88	12.95	Prospective	1
Nugroho et al. [6]	2022	Indonesia	ENE+LC	Universitas Airlangga Academic Hospital	73	1	14	0.3208 (p=0.0057)	2.151	0.7578	1.521	0.5299	Retrospective	2
Adamu et al. [12]	2022	Kano	ENE+LC	Aminu Kano Teaching Hospital		2	10	0.858 (p=0.000	67.4	15.4	0.7	0.09	Cross-sectional	1

Risk of Bias

**Figure 2:**
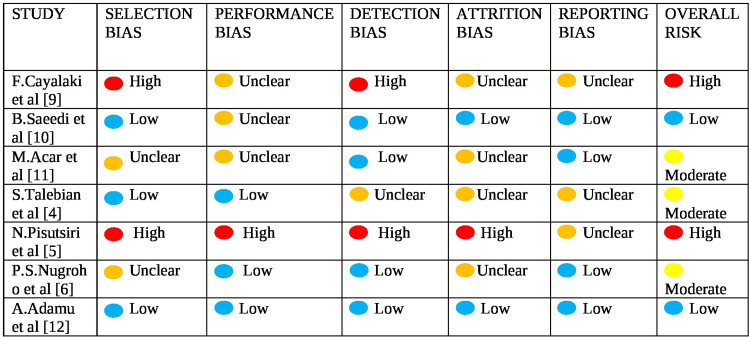
Risk of bias summary plot based on standard bias domains and a qualitative judgement

**Figure 3:**
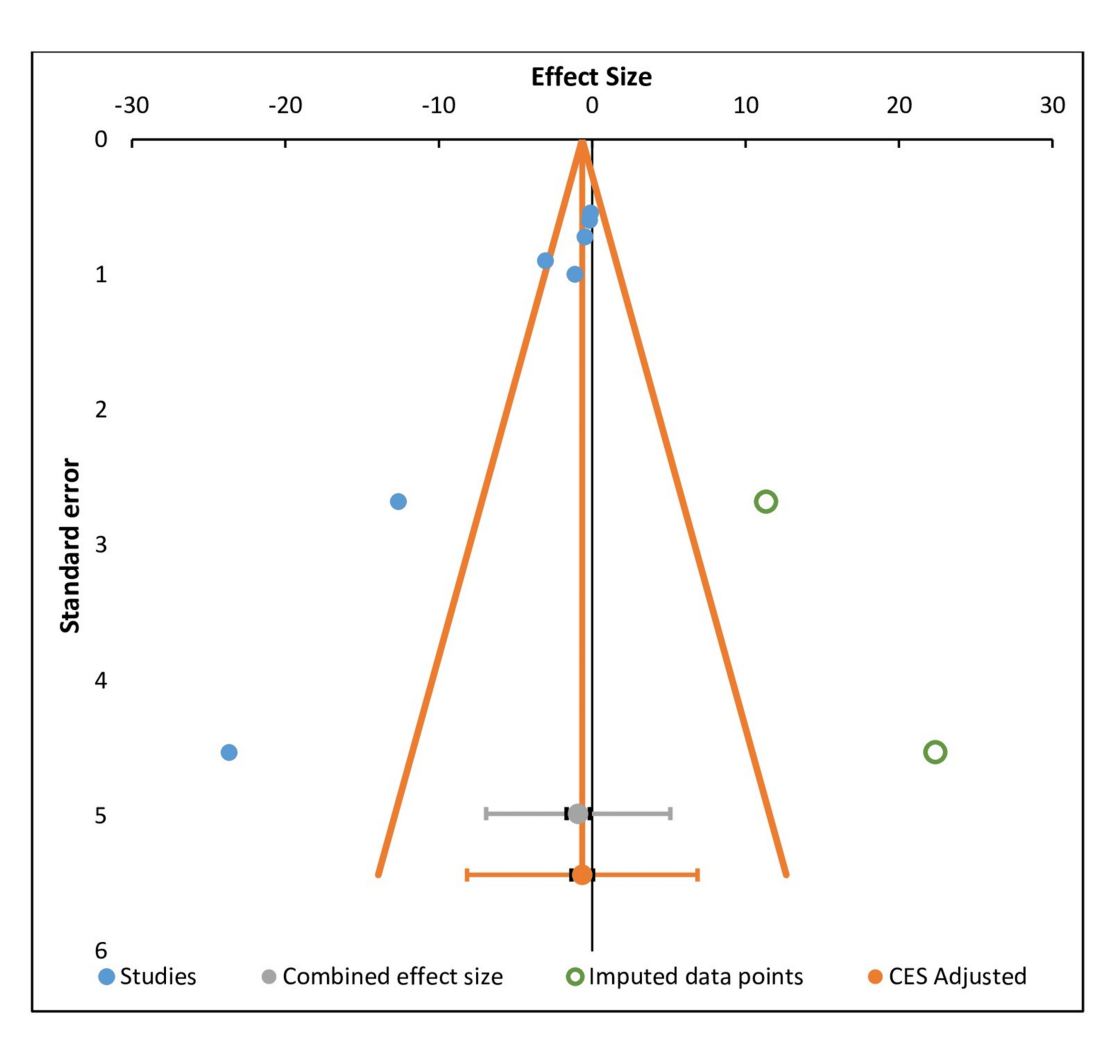
Graphical representation of publication bias CES: Combined effect size

Based on the study designs, we applied two appropriate tools to assess risk of bias: the Newcastle-Ottawa scale (NOS) for cohort studies and the Cochrane Risk of Bias tool (RoB 1) for RCTs. For RCTs, five main domains were evaluated using RoB 1: (1) selection bias, (2) performance bias, (3) detection bias, (4) attrition bias, and (5) reporting bias (Figures 2-3). For cohort studies, the NOS evaluated risk of bias based on the selection of participants, comparability of study groups, and assessment of outcomes.

The meta-analysis was conducted using RevMan 5.4 (The Cochrane Collaboration, London, GBR) and confirmed in R (meta package) for consistency. Hedges' g was used to measure the effect size in a meta-analysis, indicating the magnitude of difference between two groups or to account for small sample bias. Negative values of Hedges' g indicate a reduction in the outcome (possibly symptom severity or another negative outcome) for the intervention group compared to the control. Large negative values suggest a stronger reduction or larger effect, while values closer to zero indicate minimal or no effect.

A random-effects model (DerSimonian and Laird method) was chosen due to anticipated heterogeneity among included studies. Heterogeneity was assessed using the I² statistic, where > 50% was considered substantial heterogeneity, and τ² (tau-squared), which was used to estimate between-study variance. Confidence intervals were set at 95%, and statistical significance was defined as p < 0.05. The 95% CI provides a range within which the true effect size likely lies. If the CI crosses zero (i.e., contains both positive and negative values), it implies the result is not statistically significant at the 95% confidence level. Each study was assigned a 'weight' based on the precision of its estimate (often related to sample size and variance). Larger weights indicate a greater influence on the overall meta-analysis result. Each element is summarized in Table 3. Table 4 provides an interpretation of the studies.

**Table 3:** Terminologies for conduction of meta-analysis

Element	Meaning
Hedges' g	Standardized effect size (difference in means adjusted for sample size)
Horizontal lines	95% CIs for each study
Blue dot	Point estimate of effect size for each study
Grey dot and line at the bottom	Combined effect size and its confidence/prediction interval
Black bars (weights)	Study weights in the meta-analysis (heavily influences pooled result)

**Table 4:** Interpretation of the included studies

Study	Effect size (Hedges' g)	CI width (precision)	Likely bias/notes
Cayalaki et al. [9]	-23.67, CI ~(-40, -1)	Very wide	Low precision, likely high risk of bias or small sample size
Saedi et al. [10]	-0.17, CI ~(-2, 2)	Narrow	Precise, likely reliable
Acar et al. [11]	-3.05, CI ~(-6, -0.1)	Moderate	Moderate precision
Talebian et al. [4]	-0.12, CI ~(-5, 4)	Wide	Imprecise, low weight
Pisutsiri et al. [5]	-12.64, CI ~(-30, 7)	Very wide	High risk of bias suspected
Nugroho et al. [6]	-0.47, CI ~(-6, 5)	Wide	Imprecise
Adamu et al. [12]	-1.12, CI ~(-7, 6)	Wide	Imprecise

Interpretation of the Forest plot

**Figure 4:**
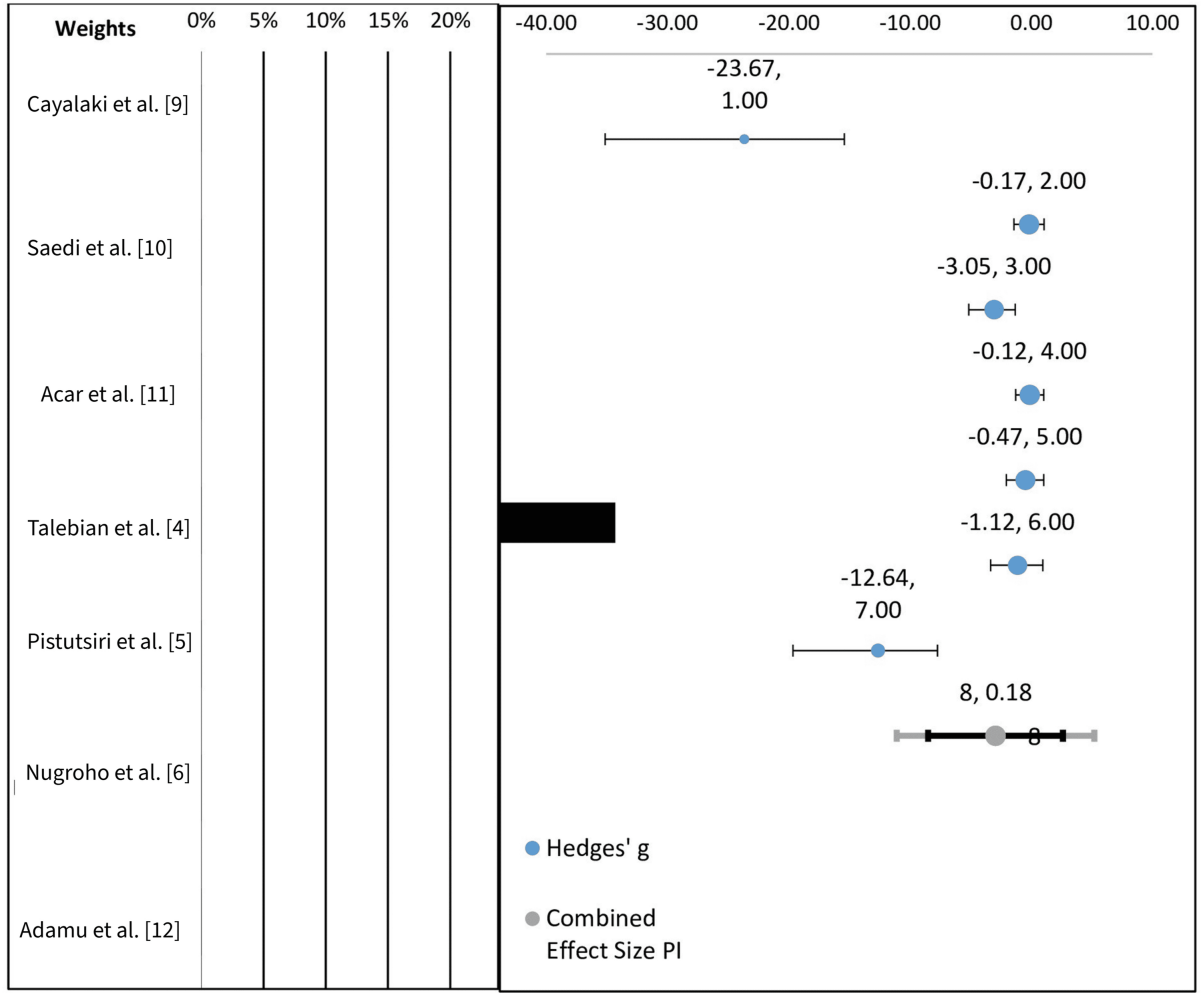
Forest plot of the representative studies

Figure 4 features the Forest plot representative of the included studies. Heterogeneity was observed. The effect sizes were inconsistent, ranging from small effects (e.g., -0.17) to very large negative effects (e.g., -23.67). This implies substantial heterogeneity, likely due to differences in study quality or populations. The studies by Cayalaki et al. and Pisutsiri et al. show extreme negative effects with wide CIs. These are likely outliers and potentially high risk-of-bias studies. The black bars representing weights are either very small or absent, which means most studies have a low influence on the pooled effect size due to imprecision or small sample sizes. However, the study by Talebian et al. contributes the most weight (visually evident from the large black bar). The bottom row shows a pooled effect of 8.00 (with CI 0.18). The pooled estimate includes 0 in its CI and therefore is not statistically significant. A prediction interval is shown (wider than the CI), indicating the expected range of effects in a new study (also includes 0).

The study by Cayalaki et al. shows an extremely large effect size, with the CI not crossing zero, indicating statistical significance. However, it has a low weight, suggesting it contributes less to the overall meta-analysis due to either a smaller sample size or higher variance. The study by Saedi et al. reports a very small effect size, and its CI crosses zero, indicating no significant effect. It carries a relatively high weight, meaning it has a substantial impact on the overall analysis. The study by Acar et al. shows a moderate negative effect with a CI that does not cross zero, suggesting statistical significance. It contributes significantly to the analysis due to its higher weight. Similar to the study by Saedi et al., Talebian et al.'s study shows a minimal effect, with a CI crossing zero, indicating no significant effect. Its high weight means it significantly influences the meta-analysis results. Pisutsiri et al.'s study shows a small effect size and has a CI that crosses zero, implying the effect is not statistically significant. It has a substantial impact due to its high weight. Nugroho et al.'s study shows a small-to-moderate effect, but the CI crosses zero, indicating no statistically significant effect. It also has a notable weight in the overall analysis. Adamu et al.'s study shows a large negative effect size, with a CI that does not cross zero, indicating statistical significance. While the effect is large, the study has a moderate weight compared to others.

Discussion

The adenoid-nasopharynx ratio (ANR) is widely used due to its simplicity and non-invasive nature. It was introduced by Fujioka et al., who stated that the ANR offers a reliable quantitative measure of adenoid size. According to them, “the ANR provides an effective method for evaluating adenoid hypertrophy using lateral neck radiographs” [13].

Furthermore, Cohen et al. confirmed the utility of ANR in clinical practice, noting that the ANR is “a good predictive tool for assessing the degree of airway obstruction in children with suspected adenoid hypertrophy” [14]. However, radiographs can suffer from certain limitations. One major drawback is the two-dimensional nature of radiographic images, which may lead to the over- or underestimation of adenoid size. Elwany et al. pointed out that “radiographs often overestimate adenoid size, especially in cases of moderate hypertrophy,” leading to diagnostic inaccuracies [15].

Nasopharyngeal endoscopy provides direct visualization of the adenoid tissue and surrounding structures, making it a highly effective tool for evaluating the extent of adenoid hypertrophy. Peedikakkal et al. noted that “endoscopy allows clinicians to assess not only the size of the adenoids but also their impact on the surrounding structures, such as the Eustachian tubes and the choanae”. This makes it particularly useful in cases where adenoid hypertrophy is suspected to contribute to more complex or severe symptoms [16].

Di Francesco et al. compared the accuracy of ANR and endoscopy, concluding that “nasopharyngeal endoscopy provided more consistent results in assessing severe adenoid hypertrophy, especially in children with obstructive sleep apnea.” The ability to observe the adenoids directly allows for a more accurate assessment of the degree of airway obstruction and can detect conditions such as nasal polyps or inflammation that may be missed on a radiograph [17].

Several comparative studies have sought to evaluate the efficacy of ANR versus endoscopic examination. Kindermann et al. found that while both methods were reliable in diagnosing moderate to severe adenoid hypertrophy, “endoscopy proved superior in detecting cases where radiographic results were inconclusive” [18]. This is consistent with findings from Yilmaz et al., who concluded that “endoscopy showed higher sensitivity and specificity in diagnosing adenoid hypertrophy, especially in cases that were difficult to assess radiographically” [19].

Wormald and Prescott also compared radiographic and endoscopic methods, noting that “while both techniques can identify moderate to severe hypertrophy, endoscopy provided a more comprehensive evaluation, leading to better-informed clinical decisions”. Given the limitations of radiographs, including the possibility of misdiagnosing the degree of hypertrophy, the authors recommended the use of endoscopy in cases where symptoms persist despite normal radiographic findings [20]. They concluded that “endoscopic examination should be regarded as the gold standard for diagnosing adenoid hypertrophy, particularly in cases where precise assessment is required.”

Clinical Utility and Limitations

While endoscopy is highly accurate, it has its limitations. The procedure is more invasive and often requires local anesthesia, making it uncomfortable for younger patients. Midulla et al. acknowledged that “although endoscopy offers greater diagnostic accuracy, it may be less feasible in very young children or in clinics without the necessary equipment and expertise” [21].

In contrast, ANR, as a non-invasive method, is quicker, more accessible, and less costly, making it an attractive option in resource-limited settings or for initial screenings. However, Elwany et al. [15] cautioned that “overreliance on radiographs, especially in cases of severe hypertrophy or persistent symptoms, could lead to misdiagnosis,” advocating for a more integrated diagnostic approach that includes endoscopy when necessary. 

## Conclusions

Both the ANR and endoscopic nasal examination are valuable tools for diagnosing adenoid hypertrophy. The ANR, due to its simplicity and noninvasive nature, is a useful first-line screening method, especially in primary care or resource-limited settings. However, its limitations, including the potential for inaccurate measurements, should prompt clinicians to consider endoscopic nasal evaluation in cases of inconclusive radiographs or persistent symptoms. Endoscopy, while more invasive, provides a more accurate and detailed assessment of adenoid hypertrophy and is particularly useful in diagnosing severe cases and planning for surgical intervention such as adenoidectomy. Ultimately, the choice between these methods should be guided by clinical presentation, availability of resources, and the need for accuracy in diagnosis.
